# Fully automated tracking of cardiac structures using radiopaque markers and high-frequency videofluoroscopy in an in vivo ovine model: from three-dimensional marker coordinates to quantitative analyses

**DOI:** 10.1186/s40064-016-1868-3

**Published:** 2016-02-29

**Authors:** Wolfgang Bothe, Harald Schubert, Mahmoud Diab, Gloria Faerber, Christoph Bettag, Xiaoyan Jiang, Martin S. Fischer, Joachim Denzler, Torsten Doenst

**Affiliations:** Department of Cardiothoracic Surgery, University Hospital Jena, 07747 Jena, Germany; Department of Cardiovascular Surgery, University Heart Center Freiburg – Bad Krozingen, Freiburg, Germany; Institute of Laboratory Animals Science, Friedrich-Schiller-University, Jena, Germany; Computer Vision Group, Friedrich Schiller University, Jena, Germany; Institute of Systematic Zoology and Evolutionary Biology, Friedrich Schiller University, Jena, Germany

**Keywords:** Radiopaque marker tracking, Cardiac mechanics, Tricuspid valve complex, Cardiac surgery, Large animal model

## Abstract

**Purpose:**

Recently, algorithms were developed to track radiopaque markers in the heart fully automated. However, the methodology did not allow to assign the exact anatomical location to each marker. In this case study we describe the steps from the generation of three-dimensional marker coordinates to quantitative data analyses in an in vivo ovine model.

**Methods:**

In one adult sheep, twenty silver balls were sutured to the right side of the heart: 10 to the tricuspid annulus, one to the anterior tricuspid leaflet and nine to the epicardial surface of the right ventricle. In addition, 13 cylindrical tantalum markers were implanted into the left ventricle. Data were acquired with a biplanar X-ray acquisition system (Neurostar R, Siemens AG, 500 Hz). Radiopaque marker coordinates were determined fully automated using novel tracking algorithms.

**Results:**

The anatomical marker locations were identified using a 3-dimensional model of a single frame containing all tracked markers. First, cylindrical markers were manually separated from spherical markers, thus allowing to distinguish right from left heart markers. The fast moving leaflet marker was identified by using video loops constructed of all recorded frames. Rotation of the 3-dimensional model allowed the identification of the precise anatomical position for each marker. Data sets were then analyzed quantitatively using customized software.

**Conclusions:**

The method presented in this case study allowed quantitative data analyses of radiopaque cardiac markers that were tracked fully automated with high temporal resolution. However, marker identification still requires substantial manual work. Future improvements including the implication of marker identification algorithms and data analysis software could allow almost real-time quantitative analyses of distinct cardiac structures with high temporal and spatial resolution.

## Background

The heart is a predominantly muscular, hollow organ that—under physiological conditions at rest—beats rhythmically 60–80/min. Under pathophysiological conditions such as e.g. myocardial infarction or cardiomyopathy, geometry, dynamics and function of the heart muscle and valves may alter significantly. To date, many of these alterations are still incompletely understood. One explanation may be that clinically available imaging modalities (such as echocardiograpy, magnet resonance imaging or computer tomography) are unable to track distinct cardiac structures. This is especially true for the thin, fast moving valve leaflets, which move with velocities up to 300 m/sec (Bothe et al. 2009).

As a consequence, an experimental imaging technique has been established where radiopaque markers are surgically implanted into regions of interest in the heart and tracked with biplane videofluoroscopy (Niczyporuk and Miller 1991). Although this technique has been proven to provide great accuracy in terms of spatial resolution, the temporal resolution was not optimal (60 Hz) and the generation of three-dimensional marker coordinates from implanted markers had several shortcomings: (a) only a small number of markers could be tracked fully automated; (b) the tracking of fast-moving structures such as valve leaflets required substantial manual correction; (c) the processing time was relatively long (e.g. a sequence containing 83 time frames and 18 markers needed 23 min of processing time); (d) images were tracked 2-dimensionally first, i.e. 3-dimensional data were obtained by reconstruction of 2-dimensional data (with the inherent risk of identification errors or the need for manual correction) (Niczyporuk and Miller 1991; Daughters et al. 1988).

To overcome these shortcomings we used a custom-built biplanar videofluoroscopy system with a temporal resolution of 500 Hz and developed computational algorithms that allow a fully automated tracking of multiple markers even on fast moving heart structures. The result of this tracking is a cloud of dots in 3-dimensional space where each dot represents a surgically placed marker (Jiang et al. 2013). For a reasonable interpretation of the markers it is, however, fundamentally important to know their exact anatomical locations on the heart.

Our first goal was, in a case study, to develop a strategy that allows assigning the exact anatomic location to each marker. Since data about the geometry and dynamics of the tricuspid valve complex are rare, our second goal was to generate quantitative data of the tricuspid valve complex from surgically implanted radiopaque markers.

## Methods

One adult female sheep was pre-medicated with ketamine (25 mg/kg intramuscularly), anaesthetized with sodium thiopental (6.8 mg/kg intravenously), intubated and mechanically ventilated with inhalational isoflurane (1.0–2.5 %).

**Fig. 1:**
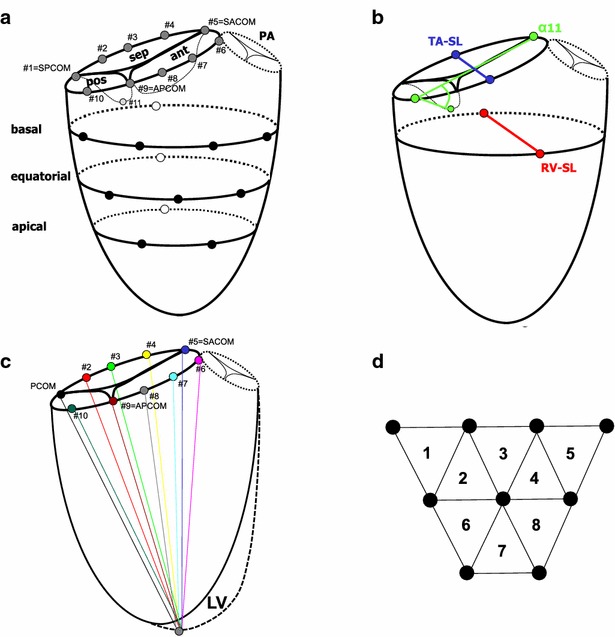
Schematic illustration of the marker array of the tricuspid valve complex (**a**): ten spherical markers were sewn to the tricuspid annulus (*dark grey circles*, #1–10), one marker was sewn to the anterior leaflet (*light grey circle*, #11), nine markers were sewn to the epicardial surface of the right ventricle (*black circles*, on a basal, equatorial and apical level) and three markers were implanted into the interventricular septum (*open circles*). To gain insight into regional tricuspid annular excursion, the distances between the left ventricular apex marker and all ten tricuspid annular markers were calculated and plotted throughout the cardiac cycle (**b**). Tricuspid annular and right ventricular septal-lateral/TA-SL and RV-SL, respectively) motion was quantified by calculating the distance between the respective marker pairs (*blue* TA-SL, *red* RV-SL). Posterior leaflet motion was determined as changes of the angle α11 (formed by a line through the tricuspid annulus and a line though the tricuspid annulus and posterior leaflet marker (*green*, **c**). Regional RV contraction was quantified by calculating the *triangular areas* formed by the respective marker triplets derived from the markers on the RV epicardial surface (**d**, *triangles* 1–7). APCOM, SPCOM and SACOM = anterior–posterior, septal–posterior and septal–anterior commissure, respectively, *ANT*, *POS*, and *SEP* anterior, posterior and septal tricuspid leaflet, *PA* pulmonary artery, *LV* left ventricle

Miniature radiopaque markers were implanted through a right thoracotomy using cardiopulmonary bypass and cardioplegic arrest. Twenty hollow silver balls (diameter: 2 mm weight: 0.003 g) were sutured to the right side of the heart: 10 markers were sewn to the tricuspid annulus (#1–10, Fig. 1a), one marker was sewn to the posterior tricuspid leaflet (#11, Fig. 1a) and nine markers were sewn to the epicardial surface of the right ventricle (four to the basal, three to the equatorial and two to the apical level, Fig. 1a). In addition, 13 cylindrical tantalum markers (length 3 mm, weight 0.009 g) were implanted into the left ventricle as described earlier in detail (Ennis et al. 2009). In brief, one marker was sutured to the LV apex and a total of twelve markers were implanted into the left ventricle on the basal, equatorial and apical level (four each) using a trocar needle. In addition, one ball and one cylindrical marker were sutured next to the marker of the anterior (#7, Fig. 1a) and septal (#3, Fig. 1a) tricuspid annulus, respectively. These markers were intended to simplify the orientation of the 3-dimensional model with the tracked markers (which was built in order to determine the specific anatomical marker locations, see “Results”).

The sheep was transferred to the catheterization laboratory immediately after surgery and studied while intubated, open-chest, and anesthetized with inhalational isoflurane (1.5–2.0 %).

Data were acquired under baseline conditions using a custom-built biplanar high-speed X-ray acquisition system (Neurostar R, Siemens AG) with a temporal resolution of 500 Hz. Along with the X-ray pictures, a video camera with identical temporal resolution (i.e. 500 Hz) was synchronized to the X-ray system in order to record data monitors displaying EKG and aortic pressure curves.

To obtain 3D reconstructions of the markers, the two cameras were calibrated using a custom-build radiopaque steel plate (Fig. 7a in Appendix) . This calibration pattern consists of 18 circular holes (each of which has a diameter of 5 mm) which are arranged in three rows (row one, two and three:five, seven and six holes, respectively). This hole arrangement allows the identification of each hole in the calibration pattern during the calibration step (Fig. 7b in Appendix). Knowing corresponding points in the calibration pattern for both views, we performed calibration of the cameras. We obtained an average back-projection error of 1.3 pixels for calibration in the sequence.

Radiopaque markers were tracked fully automated using a novel, two-staged approach for multiple marker tracking in X-ray recordings. We followed a tracking-by-detection framework for automatic multi-marker tracking. The results are 3D trajectories of individual markers over the whole sequence. Markers are firstly detected in the 2D images of both views. The detection results contain false positive and missing detections (Fig. 8 in Appendix). 3D hypotheses are constructed by triangulating the 2D detections using epipolar constraints provided by the camera calibration. Taking into account the challenges from the data (for example 2D occlusions, low contrast, missing and false positive detections) we propose a two-stage graph-based multi-marker 3D tracking approach. Primarily, we construct a weighted directed acyclic graph of 3D observations to obtain tracklets via shortest path extraction. Tracklets are short fragments of the complete trajectories and they have the highest probabilities to represent true objects. Thereafter, tracklets are linked into full tracks by another graph in a similar manner. This results in an approximation of globally optimal linking of 3D hypotheses and the intermediate tracklets while providing a flexible multi-object tracking framework. The 3D tracking results were evaluated based on the ground truth data annotated by medical experts. The quantitative assessments with respect to accuracy and precision of the tracker show that the performance of our approach is comparable to human experts. The tracking system required 4 min for processing two 3000-frame sequences each of which were implanted with 35 markers (see Ref. Jiang et al. 2013 for more details).

After marker tracking the anatomic locations of the individual markers were identified (see “Results”), one sinus rhythm heart beat was extracted and analyzed.

### Tricuspid valve leaflet opening

To quantify the opening motion of the edge of the posterior tricuspid leaflet, angle α11 was calculated as the angle in 3-dimensional space between a line through the tricuspid annulus (line between #10 and #5) and a line though the tricuspid annulus and posterior leaflet (line between #10 and #11, Fig. 1b) and plotted throughout the cardiac cycle using customized marker analysis software (Dagum et al. 2000).

### Tricuspid annular shape and dynamics

Tricuspid annular area in 3D space was calculated for each frame throughout the cardiac cycle as the sum of the areas of 10 triangles formed by consecutive adjacent marker pairs on the annulus and the annular centroid defined by markers #1–10.

In order to gain insight about shape and dynamics of the tricuspid annular height profile, annular markers were plotted in 3-D space using a right-handed cartesian coordinate system with the mid-point of all annular markers as centroid, the y-axis passing through the left-ventricular apex marker, the x-axis orthogonal to the y-axis in a plane that included the mid-septal tricuspid annular marker (#3, Fig. 1a) and the z-axis orthogonal to the x–y plane pointing anteriorly. In addition, the orthogonal distance from each annular marker to the least-squares tricuspid annular plane was calculated. Both, marker coordinates and annular height profiles were plotted when the tricuspid annular area reached its minimum (MinTAA) and maximum (MaxTAA) at the heartbeat analyzed.

Tricuspid annular septal-lateral diameter was calculated from markers #3 and #8 (Fig. 1a).

### Global and regional right ventricular function

The systolic excursion of the tricuspid annular plane (TAPSE, defined as distance change between the anterior part of the tricuspid annulus and the left ventricular apex during systole) is a common parameter to quantify global right ventricular function using echocardiography (Rudski et al. 2010). To gain insight into regional tricuspid annular excursion, the distances between the left ventricular apex marker and all ten tricuspid annular markers were calculated and plotted throughout the cardiac cycle (Fig. 1c). Furthermore, global and regional epicardial shortening of the right ventricle was assessed. Regional RV shortening was measured by calculating the areas of all nine right ventricular markers (triangles 1–8, Fig. 1d) throughout the cardiac cycle (regional right ventricular surface area). Total right ventricular surface area was defined as sum of all eight triangles. Similarly, total left ventricular surface area was estimated from summing the triangles of the left ventricular markers.

Right ventricular septal-lateral diameter was calculated from the marker sutured to the midline of the right ventricle on the basal level and the corresponding marker at the ventricular septum (Fig. 1b).

The animal experiment adhered to relevant regulations and was approved by the federal state of Thuringia, Germany.

## Results

### Marker identification

**Fig. 2:**
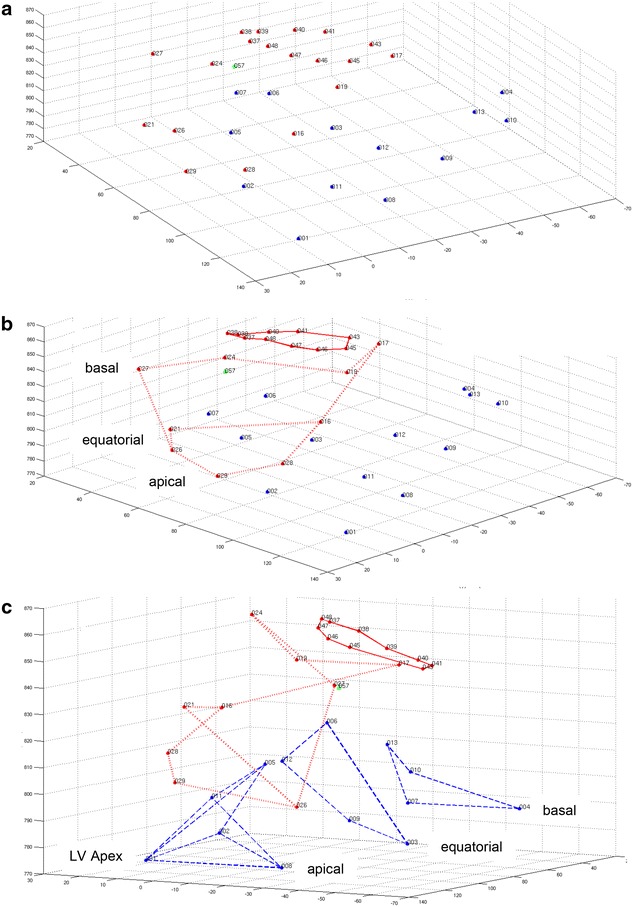
Three-dimensional model of a single frame from all markers. Cylindrical markers were manually separated from ball markers, thus allowing to distinguish right heart (*red dots*) from left heart (*blue dots*) markers (**a**). Using video loops constructed of all recorded frames allowed identification of the fast moving leaflet marker (*green dot*, **a**). Afterwards tricuspid annular, leaflet, as well as right and left ventricular markers were identified and connected with virtual lines (tricuspid annulus: *red line*, right ventricle: *red dotted line*, left ventricle: *blue dashed line*) using a Matlab visualization tool (**b**, **c**). *LV* left ventricle

In a first step a 3-dimensional model of a single frame containing all tracked markers was built. In this model all markers appeared as simple dots. This model was then compared with the raw X-ray images of both cameras and dots resulting from cylindrical/ball markers were identified. This identification allowed to distinguish right from left heart markers (red dots represent right heart markers, blue dots left ventricular markers, see Fig. 2a). Afterwards, the fast moving leaflet marker was identified by using video loops constructed of all recorded frames (green dot, Fig. 2a–c). Lastly, rotation of the 3-dimensional model and the use of the two guide markers (see “Methods”) allowed the identification of tricuspid annular as well as right and left ventricular markers. Markers of each anatomical entity were connected with virtual lines using a Matlab visualization tool (Fig. 2b, c).

### Quantitative marker analyses

**Fig. 3:**
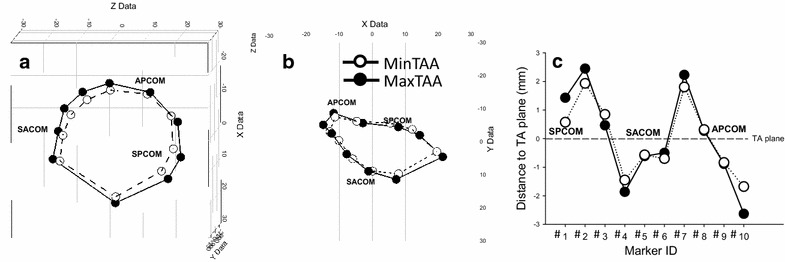
Three-dimensional coordinates of the ten tricuspid annular (TA) markers (**a**, **b**): and marker distances to the TA plane (**c**) at the timepoint of maximum and minimum TA area (*closed* and *open dots*, respectively, see Fig. 1 for definition aof marker IDs). APCOM, *SPCOM* and *SACOM* = anterior–posterior, septal–posterior and septal–anterior commissure, respectively

Figure 3a, b show the coordinates of the tricuspid annular markers at MaxTAA (closed dots) and MinTAA (open dots). Figure 3c shows the tricuspid annular height profile at MaxTAA (closed dots) and MinTAA. The tricuspid annulus is saddle shaped with its highest points in the septal and anterior region and undergoes significant changes in shape and size during the cardiac cycle. The tricuspid annular height profile flattens during annular contraction (MinTAA) and increases with annular relaxation (MaxTAA, Fig. 3c).

**Fig. 4:**
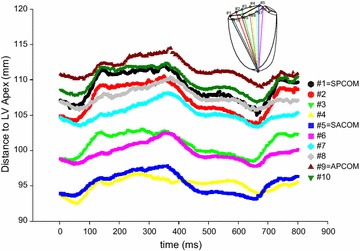
Distances between different tricuspid annular segments to the LV apex. The change in distance between the mid-anterior tricuspid annular marker (#8) and the LV apex is a frequently used parameter to quantify right ventricular contraction

Figure 4 illustrates alterations of the distance between different tricuspid annular segments to the LV apex during the cardiac cycle. The amount of distance change during a heartbeat appears similar for each of the ten tricuspid annular segments.

**Fig. 5:**
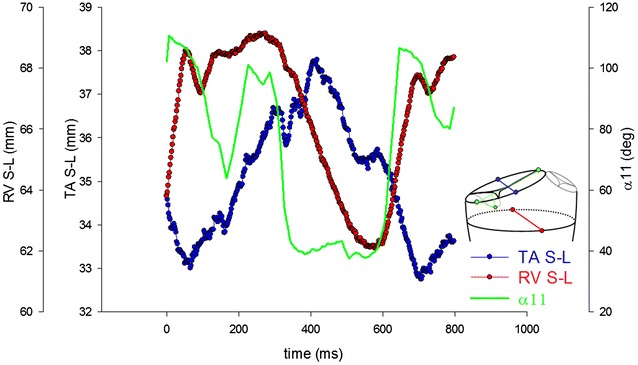
Temporal relationship between tricuspid annular contraction (represented by the change in tricuspid annular septal-lateral diameter (TA S-L), right ventricular contraction (represented by the change in right ventricular septal-lateral diameter (RV S-L) and closing motion of the posterior tricuspid leaflet (represented by the change in α11, see “Methods” for details)

The temporal relationship between tricuspid annular contraction, right ventricular contraction and closing motion of the posterior tricuspid leaflet is shown in Fig. 5. Right ventricular contraction (decrease in TA S-L) occurs at approximately 250 ms and is associated with a closing motion of the tricuspid posterior leaflet (sudden decrease in α11). Tricuspid annular contraction (decrease in TA S-L) occurs later in the cardiac cycle, i.e. after approximately 420 ms.

**Fig. 6:**
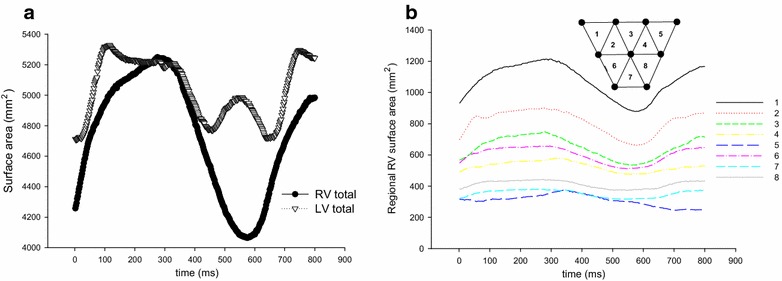
Alterations in total left and right ventricular (LV and RV total, respectively) surface area (**a**) as well as regional alterations in right ventricular surface area (**b**)

**Fig. 7:**
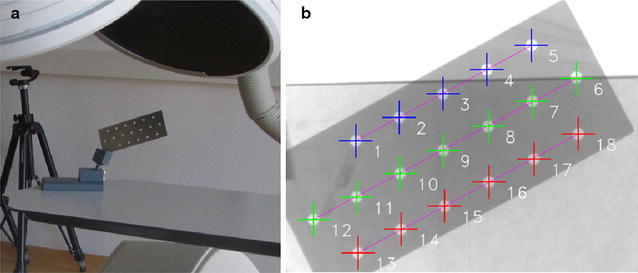
To obtain 3D reconstructions of the markers, the two cameras were calibrated using a custom-build radiopaque steel plate (**a**). This calibration pattern consists of three rows of 5 mm holes. This hole arrangement allows the identification of each hole in the calibration pattern during the calibration step. **b** Shows the identification of the holes

**Fig. 8:**
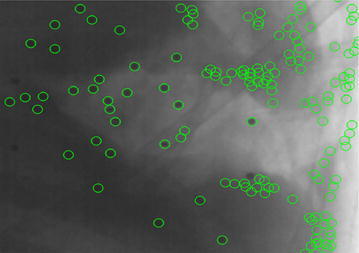
Markers are firstly detected in the 2-D images of both camera views. The figure shows an example of marker detections in the left camera image. The detection results contain false positive and missing detections

Figure 6a, b show alterations in left and right ventricular surface area (Fig. 6a) as well as regional alterations in RV surface area (Fig. 6b). The decrease in total surface area of right and left ventricle occurs simultaneously at 300 ms. While the total surface area of the right ventricle decreases by an amount of 1000 mm^2^, the total left ventricular surface area decreases only by 500 mm^2^. The regional contraction of the right ventricle appears greatest in the basal region far from the pulmonary outflow tract (triangles 1–3, Fig. 6b) and less in the equatorial regions or the regions close to the pulmonary outflow tract (triangles 4–8, Fig. 6b).

## Discussion

The method of radiopaque marker tracking used in this experimental setup builds on previous techniques by improving temporal resolution and increasing full automation of marker tracking, which decreases processing time and the risk of errors due to manual correction or misidentification. The result of this tracking approach is, however, a cloud of dots in 3-D space where each dot represents a surgically placed marker. In a case study we manually identified the anatomical position of each marker and performed quantitative data analyses.

The experimental imaging modality of radiopaque marker tracking is, in contrast to current clinical imaging modalities (such as e.g. echocardiography, CT or MRI), capable of tracking distinct cardiac structures. Other experimental imaging modalities to track distinct cardiac structures exist [e.g. sonomicrometry (Gorman et al. 1996)]. Radiopaque marker tracking has, however, the advantage that more tantalum markers can be placed than sonomicrometry transducers, and that tantalum markers can label more delicate, fast-moving structures, because they do not have wires attached (Gorman et al. 1996). We therefore focused on advancing the method of radiopaque marker tracking.

While a novel marker tracking approach (allowing faster, more precise tracking of multiple, even fast moving markers) has been published previously (Jiang et al. 2013), the steps from marker tracking towards an identification of anatomical marker locations have not been described. Although the steps towards a full identification of all markers were time consuming and could only performed manually (e.g. separation from cylindrical from ball markers), they allowed a safe determination of the marker locations. Future improvements could include automated separation between ball and cylindrical markers as well as computational algorithms that allow an automated identification of ventricular, annular and leaflet markers. The use of markers with different shapes could further simplify automated marker identification. It should, however, be considered that any shape other than spherical becomes less optimal from the perspective of marker tracking (the 2-dimensional shape of a ball is independent of the angle of the X-ray camera).

In order to obtain quantitative results from our marker tracking we used a software that was customized for this particular experimental setup (Dagum et al. 2000). In case this (or a similar) software can be linked directly to marker tracking and identification, this experimental methodology could allow almost real-time quantitative analyses of distinct cardiac structures with very high temporal and spatial resolution.

The software described above can potentially integrate simultaneously acquired hemodynamic and electrocardiographic digital data. These data were, however, not digitally acquired in our experimental setup, but from a video camera that was synchronized with the X-ray system (i.e. analog). First, this method allows only an inaccurate acquisition of hemodynamic data and the data were therefore not used for our data analyses. Second, the hemodynamic state for each marker frame needs to be converted into digital data. This step does not only require substantial work, but is a source for conversion errors and, thus, imprecision of this methodology. In the future, direct digital acquisition of EKG and hemodynamics synchronous to the X-ray cameras is desirable.

Data from radiopaque marker tracking of the right heart have been shown previously (Moon et al. 1994; Chin et al. 1989). In agreement with other studies we found a saddle shape of the tricuspid annulus (Ton-Nu et al. 2006). However, dynamics of the tricuspid annular height profile during different time points of the cardiac cycle have not been described. While an increase in saddle shape is described during mitral annular contraction (Nguyen et al. 2008), the tricuspid annulus flattens during contraction in our study. In case these data hold true in other experiments, they may not only provide important novel information about the physiological shape of the tricuspid annulus, but may also help to improve surgical repair approaches of the diseased tricuspid valve (which include the implantation of a physiologically shaped annuloplasty ring).

The TAPSE is a common parameter to quantify global right ventricular function using echocardiography (see “Methods”) (Rudski et al. 2010). While the assessment of the excursion of the anterior tricuspid annular segment is relatively straightforward and reproducible, other tricuspid annular segments (septal, posterior) in combination with the LV apex are more difficult to visualize. However, a more detailed insight into alterations of the TAPSE in different annular regions, e.g. during the cardiac cycle or in a diseased state, may help to further improve the understanding of a malfunctioning right ventricle and annulus.

The temporal pattern of tricuspid valve closure, tricuspid annular and right ventricular contraction has, also due to shortcomings of current imaging modalities, never been investigated before. Right ventricular contraction and the closing motion of the tricuspid anterior leaflet occur simultaneously, whereas the reduction of the tricuspid septal-lateral diameter occurs about 150 ms later in the cardiac cycle when the leaflet is closed. It could therefore be speculated that the tricuspid leaflets have a sail like function [as suggested for the posterior leaflet of the mitral valve (Bothe et al. 2009)], where annular shortening occurs secondary to right ventricular contraction rather than being the result of an active annular contraction. If true, annular repair approaches should focus more on preserving valve function rather than preserving tricuspid annular contraction.

Furthermore, alterations in left and right ventricular surface area as well as regional alterations in RV surface area are shown. The increase in LV surface area during ventricular contraction after an initial decrease could result from wall motion abnormalities due to the acute open-chest experimental setup and is not in line with other studies which describe a continuous decrease of LV surface area during systole (Ourselin et al. 2013). The decrease in total surface area of right and left ventricle occurred simultaneously in this case study. The investigation of temporal time patterns between left and right ventricular contraction is important, since an asynchrony has been described to aggravate valve dysfunction or cardiomyopathy (Bordachar et al. 2003). This experimental method would allow for the detailed assessment of changes in synchrony between right and left ventricular contraction in several diseased settings or of the effects of a cardiac resynchronization therapy. Furthermore we found a greater reduction of the total right ventricular surface area than of the left ventricular surface area. Left ventricular systolic output (i.e. decrease in left ventricular volume) is mainly driven by wall thickening and left ventricular torsion (Ennis et al. 2009; Rodriguez et al. 2004). Since the right ventricular myocardium is significantly thinner than that of the left ventricle, a decrease in epicardial surface area could be of a greater importance than wall thickening, which also suggests fundamentally different myocardial contraction mechanisms between left and right ventricle. In addition, we found regional heterogeneities with respect to alterations in right ventricular surface area. A better understanding of physiological right ventricular contraction patterns or of changes in regional contraction in the case of right ventricular heart failure could help to improve therapies targeting a mechanical support of the right ventricle.

### Limitations of the analyses

This case study represents data from one sinus beat in one animal under open chest conditions immediately after cardiopulmonary bypass and cardioplegic arrest. More data are needed to proof the reproducibility of our marker identification strategy and to allow a meaningful interpretation of the quantitative data.

In conclusion, the steps of identifying anatomical marker locations and performing quantitative analyses from radiopaque cardiac markers—which have been tracked using high-speed biplanar videofluoroscopy and novel tracking algorithms—proved to be feasible in this case study, but still require substantial manual work. Future goals include the implication of novel marker identification algorithms, the establishment of simultaneous digital acquisition of hemodynamic data and the implication of quantitative marker analysis software. These improvements could allow, for the first time, almost real-time quantitative analyses of distinct cardiac structures with high temporal and spatial resolution. The results of our preliminary data analyses suggest that this experimental imaging methodology could significantly help to improve our understanding of the physiological geometry and dynamics of the tricuspid valve complex and treatment strategies focusing on tricuspid valve dysfunction or right ventricular heart failure.

## Appendix

See Figs. 7 and 8.
